# A Brief Paper on the Comparative Effects of Alcohol and Tobacco, on the Human System

**Published:** 1871-03

**Authors:** N. S. Davis

**Affiliations:** Chicago, Ill.


CHICAGO

MEDICAL EXAMINER:

N. S. DAVIS, M.D., EDITOR.

F. H. DAVIS, ASSISTANT EDITOR.

VOL. XII. MARCH, 1871. NO. 3.

### Original Contributions.

### A BRIEF PAPER ON THE COMPARATIVE EFFECTS OF ALCOHOL AND TOBACCO, ON THE HUMAN SYSTEM.

By N. S. DAVIS, M.D., Chicago, Ill.

Prepared by the request of the Chicago Medical Society, and read at the Regular Meeting, Dec. 26, 1870.

There are but few questions connected with the social and economical interests of the human family more important than those which relate to the influence of alcoholic drinks and tobacco. Whether we consider the vast expenditure of money occasioned by their use, the impoverishment they help to perpetuate among all classes; and their indirect effects upon the sanitary condition of individuals and communities; or whether we study their more direct influence over the moral, social, and physical condition of the consumer, we shall be overwhelmed with the magnitude of the subject, and astonished at the tenacity with which popular errors are perpetuated from generation to generation. It is no part of our present purpose, however, to enter upon so broad a field of inquiry. From the paper read at a previous meeting of the Society on the effects of tobacco, and the discussions thereon, I infer that the task which the Society intended to impose on me for the present occasion was, simply to present my views of the *modus operandi* of alchohol and tobacco, when taken into the human system, and how far they mutually co-operate with or antidote each other. Hence I shall state, in as few words as possible, the results of the strictly scientific investigations concerning those agents, which have been made in Europe and America, during the last half century.

From all I have been able to find in our literature, or to develop from my own inquires in relation to the influence of alcohol, the following propositions appear to be fully established:

*First.* Numerous chemical analyses of the blood and different tissues, made by different experimenters, show that when alcoholic drinks are taken, the alcohol enters the blood and permeates with it every part of the body.

This position is now acknowledged to be correct by all classes of observers.

*Second.* An equally reliable series of experiments have shown that the alcohol undergoes no chemical change in the system, but is eliminated through the excretory organs, more especially the lungs and kidneys, generally within a few hours after it is taken.

This position has long been disputed, but it was finally fully established by the results of the, well-devised and carefully-executed, experiments of Lallemand, Perrin, and Duroy.

*Third.* While the blood is circulating through the system, the alcohol diminishes the sensibility of the brain and nervous system in the same manner as other anæsthethics, and also retards the active changes in all the tissues; and consequently diminishes the sum total of eliminations or excretions in a given period of time.

The numerous and patient experimental investigations of Prout, Sandras and Bouchardet, Boker, Hammond, and others, have removed all doubts in regard to the truth of this proposition.

*Fourth.* By diminishing the atomic changes in the tissues of the body and the sensibility of the nervous system, the alcohol by its presence also diminishes the temperature, the strength, and the power of endurance. That its presence in the system reduces the temperature, was first fully established by the results of a series of experiments performed by myself in 1850, some of which I repeated in 1867. These experiments consisted in testing the actual temperature of the body every half hour, with a delicately-graduated thermometer, for three hours after a moderate drink of alcoholic liquor. The tests were applied to both wine and whiskey.

These results are confirmed by the observations of Magnus and others in Europe. That the presence of alcohol directly diminishes the strength and power of endurance, is proved, not only by the foregoing scientific investigations, but also by a large number of carefully-observed facts in relation to the results of labor, in civil and military life, and by the statistics of sickness and mortality in all parts of the civilized world.

I am not aware that any well-informed writers, at the present time, call in question the correctness of the first and third propositions here stated; and hence we may regard them as expressions of established truths. The second and fourth, however, have thus far failed to command the assent of a large class of professional men and chemico-physiological writers, and consequently still elicit contradictory expressions of opinion. The second proposition declares that the alcohol undergoes no chemical change in the system, but is eliminated through the excretory organs, like other substances incapable of assimilation.

This proposition has met with the most persevering opposition. For a long period of time alcohol was almost universally regarded as tonic and nutritive. To use the emphatic language of another, it was represented to be "the conservator of strength in manhood, and the *milk* of age." In the rapid development of organic chemistry as a branch of science, Liebig and his followers, classing alcohol with the hydro-carbons, on purely theoretical grounds, declared it to be pre-eminently *respiratory food*. By which they meant that it united with the oxygen in the system, and was resolved into carbonic acid and water with the evolution of caloric, thereby explaining its supposed efficacy in sustaining the temperature and activities of the system. But the experiments of Prout of England, Sandras and Bouchardet of France, and Boker of Germany, so clearly demonstrated that the presence of alcohol in the system diminished, not only, the elimination of carbonic acid and the absorption of oxygen, but retarded all the organic or atomic changes in the system, that the doctrine of Liebig was speedily abandoned. In its place, however, came another equally theoretical and more unphilosophical, namely, that inasmuch as alcohol retards the metamorphosis of tissue by retarding the atomic changes, it becomes "indirect" or "*accessory food.*" This is the doctrine more or less distinctly taught by Austie, Johnson, Hammond, and other eminent writers of the present day. They assume that if the presence of a given quantity of alcohol diminishes the sum total of organic changes and eliminations to the extent of one pound, for example, in twenty-four hours, it is just equivalent to that much food or new matter added. In other words, to keep up the popular idea that alcohol is in some way *food* to the human system, the position is clearly assumed that to retard tissue metamorphosis is equivalent to tissue nutrition. It seems hardly possible that men of eminent attainments in the profession should so far forget one of the most fundamental and universally recognized laws of organic life, as to promulgate the fallacy here stated. The fundamental law to which we allude is, that all vital phenomena are accompanied by and dependent on molecular or atomic changes; and whatever retards these retards the phenomena of life; whatever suspends these suspends life. Hence to say that an agent, which retards tissue metamorphosis, is in any sense *food*, is simply to pervert and misapply terms.

To be consistent we must apply the same rules of inquiry to the investigation of the effects of alcohol, that we do to all other substances. When various salts of potassa, soda, mercury, arsenic, strychnine, etc., are taken into the system, and by appropriate analyses and tests they are found to have entered the blood, permeated the tissues, and reappeared in one or more of the excretions without chemical change, it is universally regarded as sufficient to prove that they are agents foreign to the animal economy, incapable of assimilation, and therefore medicines as distinguished from food. Apply the same rules to alcohol, and it must be placed in the same category.

For all concede the fact that it has been detected abundantly in the blood, the tissues, and nearly all of the excretions. The pretext that because no one has actually collected from the various eliminations, within a given time after taking alcohol, the exact quantity taken, a part of it may still be used up as some kind of nutrient in the system, is merely begging the question.

I am not aware that any chemist has collected from the excretions or tissues the exact amount of soda, potassa, mercury, arsenic, or strychnine, that may have been taken into the system at a given time. But does any one, on that account, claim that some part of these substances may, after all, act as *food*, either direct or *indirect?* If we allow the same rules of investigation, and the same laws of induction to apply in arriving at conclusions concerning the action of alcohol, as we do in relation to all other agents we must regard the correctness of the second proposition as fully established.

The assertion in the fourth proposition that the presence of alcohol reduces the temperature of the system, though still doubted by some, is so generally conceded by writers at present, that I shall only refer to two or three additional items of proof that have come under my observation within the last few days. Dr. Rabateau has recently published in the *Union Medicale of France*, a paper giving his researches upon this subject, of which the following is stated as one of his conclusions, *viz.:* "Alcohol *lowers* the temperature, retards the organic combustion, and consequently diminishes the carbonic acid and urea. Its action on the blood globules is the same as that of arsenic and oxide of carbon. It is *antipyretic* and *antiphlogistic.*

In a recent number of the New York *Medical Journal*, Dr.

P. DeMarmon reports three cases of the poisoning of children by whiskey. The first case was a boy five years of age, who took a tumbler of whiskey at six o'clock in the morning. At nine o'clock P.M., fifteen hours after the whiskey was taken, the temperature, as taken in the axilla, was 93½° F. In four hours more the child died.

The second case was that of a girl aged five years, who took a tumblerful of whiskey-and-beer, at four o'clock P.M. She was seen by the doctor two and a half hours after, and found to be profoundly unconscious, having had involuntary discharges, anl her temperature was reduced to 94° F. She was placed on appropiate treatment, and finally recovered.

The third case was a boy eight years of age, who took a quantity of whiskey at eight o'clock in the morning; soon became insensible and died in twenty-one hours, and his temperature was not ascertained.

Dr. N. S. Cheever of the Michigan University, has recently reported the results of twenty-eight different observations concerning the effects of various alcoholic drinks on the temperature of different individuals, from which he deduces the following propositions:

"1. That non-poisonous doses of alcoholic liquors have but little, if any, influence upon the body temperature of man.

"2. That this influence, when exerted, is not uniform, but varies with individuals and liquors.

"3. That this influence alone is not sufficient to indicate or contraindicate the use of these agents in any case."

In the limited number of cases in which the taking of alcoholic drinks has been followed by an appreciable increase of temperature, we think a careful examination will show that such increase has been either limited to the first twenty or thirty minutes after the drink was taken, or to those cases in which food was given in conjunction with the alcohol. In the first, the increase of temperature is caused by the direct contact with and anæsthetic effect on the gastric branches of the pneumogastric nerves, and is limited to the few minutes that intervene before the alcohol has had time to reach the arterial side of the circulation, or to permeate the tissues generally. A similar temporary increase of temperature has been found to follow cutting or irritating the pneumo-gastric nerves in the neck. The fact that the increase of temperature is claimed to be observable within five minutes after the alcohol is taken, and generally continues less than thirty minutes, is sufficient to show that it is owing to the local effect on the gastric nerves, and not to the presence of the agent in the blood. That a slight increase of temperature is observable after taking alcoholic drink in connection with milk, egg, or other food, is undoubtedly true. But the same, or even greater increase would be found to occur after taking the same food without the alcohol. More than twenty years since, we proved by numerous careful experiments, that the temperature of the human system is always increased from 0.5 to 2° F. during the period of active digestion after taking ordinary food. To judge accurately of the true physiological effects of alcoholic drinks on the functions of the human system, they should be taken with the individual at rest, the stomach unoccupied by food, the atmospheric temperature equal, and the observations continued through sufficient time, to allow the agent to complete its work by absorption, circulation, and elimination. When these conditions have been observed, we have found no evidence that alcoholic liquors increased the temperature, but, on the contrary, that they caused diminution, in small doses, of from 0.5 to 1.5° F., and in poisonous doses from 3° to 5° F.

That the presence of alcohol directly diminishes the strength and power of endurance, is proved, not only by the foregoing scientific investigations, but also by a large number of carefully observed facts in relation to the results of labor, in civil and military life, and by the statistics of sickness and mortality.

Twelve or fifteen years ago an article appeared in the *British and Foreign Medico-Chirurgical Review*, embracing a large amount of statistical information on this subject. At one place in England, where a large amount of brick-making is carried on, and where the amount of each man's work, the number of days lost by sickness or otherwise, and the deaths, were made matters of record, the rules of the service allowed to every man a mug of beer at each meal.

But there were among the workmen quite a number who wholly abstained from the use of the beer or any other intoxicating drink.

An examination of the record showed that the average amount of work done, per annum, by the beer-drinkers was a large percentage less than that done by those who wholly abstained, while the number of days lost by sickness was greater. The same relative results have been shown in reference to every other species of manual labor where records have been kept sufficient to afford a comparison. The article to which we have just alluded contained some interesting items from the reports of the Registrar-General of the British Army. At that time the soldiers of the army in the East Indies were allowed regular daily rations of whiskey; but there were some companies of pledged teetotalers, who faithfully declined the ration. On examining the details of the regular reports concerning sickness and mortality in that army, it was found that the ratio of sickness and mortality among the teetotalers was from five to ten per cent. less than for the rest of the army.

The history of our own army affords some facts of importance on this subject. Dr. Mann, a surgeon of excellent authority, who served with the army of the Revolution, says: "At that period, during the revolutionary war, when the army received no pay for their services, and possessed not the means to procure *spirits*, *it was healthy.* The Fourth Massachusetts regiment, at that eventful period of which I was surgeon, lost in three years, by *sickness*, not more than five or six men. It was a time when the army was destitute of money. During the winter of 1779–80, there was only one occurrence of fever in the regiment, and that was a pneumonia of a mild form. It was observable in the last war, from December, 1814, to April, 1815, the soldiers at Plattsburg were not attacked with fevers as they had been the preceding winters. The troops during this period were not paid; a fortunate circumstance to the army, arising from the want of funds."

These may be said to be only negative results, but unhappily the history of the recent war for the suppression of rebellion furnishes some examples of a positive character. While General Cass was Secretary of War, several years since, the whiskey rations were discontinued in the United States Army, and coffee and sugar substituted. This has been the army regulation ever since. The army of the Potomac, in the spring of 1862, was subjected to great hardships in labor, and exposed to the extremely wet and malarious region of the Chickahominy. There was consequently much sickness and suffering. Under these circumstances, the commanding general issued an order on the 19th of May, allowing every officer and soldier one gill of whiskey per day, half to be served in the morning and half in the evening.

The results were so manifestly injurious to the sanitary condition of the army, that in just thirty days the order was fully countermanded by the same general. Concerning this experiment in the Army of the Potomac, Dr. Frank H. Hamilton, one of the most eminent surgeons serving with that army, says: "It is earnestly desired that no such experiment will ever be repeated in the armies of the United States. In our own mind, the conviction is established by the experience and observation of a life that the regular routine employment of alcoholic stimulants by man in health is never, under any circumstances, useful. We make no exceptions in favor of cold, or heat, or rain, nor, indeed, in favor of old drinkers, when we consider them as soldiers."

For these and other facts bearing on this subject, see Dr. Hamilton's valuable work on Military Surgery, from page 70 to 75. Further facts of a striking and perfectly authentic character, bearing on this subject, may be found in one of the volumes of "Medical Inquiries and Observations, by Benjamin Rush, M.D.," in a chapter devoted to a comparison of the diseases prevalent in Philadelphia during ten years previous to the revolutionary war, with those of a similar period after the close of that war. Indeed, it were easy to fill a volume with facts and statistics showing that in every relation of human life, the use of alcoholic drinks diminishes man's capacity to endure both mental and physical labor; increases his predisposition to disease; and shortens the average duration of life. And, although we have had our attention directed to this subject for thirty years, we have not found, either in the records of medicine or of general literature, a single statistical item calculated to prove the contrary. We have seen an abundance of *opinions* expressed of an adverse character. But *opinions* are not facts.

It is very common to hear that some sick or injured person has been kept up, or "kept alive," on brandy, or whiskey, or wine, for several weeks. But do those who make the assertion have any reliable means of knowing whether the sick person was actually kept alive by the alcoholic potion, or whether he lived in spite of it? The eminent Dr. Todd testified strongly to the sustaining and beneficial influence of alcoholic drinks in the low forms of fever, yet statistics show that in the London fever hospitals, with which he was connected, the ratio of mortality increased *pari passu* with the increased use of alcoholic drinks as remedies. The able corps of medical attendants on the Bellevue and Emigrant Hospitals in New York also bore decided testimony to the utility of these liquors in the treatment of the same forms of fever, and used them largely. But the mortality was one in every five or six cases treated. The same fevers placed in tents with plenty of fresh air and nourishment, without a drop of alcoholic drinks in their treatment, gave a mortality of only one in seventeen.

The statistics of almost all epidemics show that those addicted to the use of intoxicating drinks are much more liable to be attacked than those who abstain; and of those who are attacked, a larger proportion die. If alcohol takes no part in nutrition, but passes through the blood, disturbing the nervous sensibility and retarding the organic changes, some may be ready to ask why habitual drinkers of wine and beer grow fat and increase much in weight. We answer, that the presence of alcohol in the blood not only retards atomic changes in the tissues, but it diminishes the amount of oxygen taken in through the lungs. Consequently, the carbonaceous elements of the blood and tissues do not become oxidated as rapidly as when the alcohol is not present, and they accumulate in the form of *fat*. And this process is sometimes carried so far that the heart, liver, and kidneys undergo more or less fatty degeneration, constituting incurable forms of disease.

The increase of bulk and weight in these cases is not from an increase of natural nutrition, but a slow accumulation of hydro-carbonaceous material from retarded metamorphosis. The individual fattened under such influences invariably diminishes in physical activity and power of endurance, in proportion to the increase of weight.

The simple logical deduction from all the carefully observed facts in relation to this subject, is, that alcoholic drinks, when taken into the human system, circulate in the blood as foreign agents, and until eliminated or cast out through the excretory organs, produce a temporary exhilaration of the mental faculties, accompanied by diminished sensibility, temperature, strength, and atomic changes. And when these effects are often induced, they result in a more or less permanent morbid or diseased condition of the gastric branches of the pneumogastric and ganglionic nerves, by which morbid impressions are transmitted to the censorium, constituting what is styled an appetite or craving for stimulating drinks. But the state here alluded to is not a mere appetite dependent on a morbid condition of the gastric nerves. On the contrary, the alcohol in the blood, permeating all the tissues, by its strong affinity for albumen, modifies the play of vital affinity in such a manner as to retard those atomic or molecular changes which constitute nutrition, disintegration, and secretion. As soon as the direct anæsthetic effects of the alcohol have ceased by a failure to renew the dose, in persons whose tissues have been habituated to its impressions, there comes a general feeling of uneasiness, an indescribable restlessness, often a sense of exhaustion; or, to use a phrase common to such persons, they "feel as though they could not live" without something to sustain them. It is this interference with the natural molecular changes, and the consequent morbid condition of the properties of the tissues generally, that so irresistibly impels the habitual drinker to continue his ruinous practice, more than a mere appetite or relish for the liquor itself.

The influence of alcohol on the properties and molecular changes of the tissues, leads to three ultimate results, according to the quality and quantity taken in a given period of time. If distilled spirits are used freely and continually, the alcohol so rapidly retards metamorphosis, and lessens the sensibility of the gastric nerves, that all appetite for food is lost. The processes of digestion and assimilation are so far suspended that the brain, as well as the other structures of the body, fails to receive a sufficient supply of new atoms to enable it to perform its functions, and in from two to four weeks, the individual is arrested in his career, by the supervention of delirium tremens. If the same kind of drinks are used daily, but in more moderate quantity, a certain amount of appetite and digestion is retained. Assimilation and nutrition continue, but in a more or less perverted manner. The blood becomes slowly impoverished of its nitrogenous nutritive elements; the nervous and muscular structures become weakened by the deposit in them, of fat granules, instead of their own proper substances; the liver, or kidneys, or both, undergo the same fatty degeneration; and thus, in a few years, we have developed that peculiar condition so well known as *chronic alcoholism*, or the alcoholic diathesis. But if fermented drinks, such as ale, porter, wine, and beer are used, with only now and then a small quantity of distilled spirits, the quantity of alcohol in the blood will not be sufficient to cause impoverishment of that fluid, or rapid changes in nutrition and waste. Its presence, however, will so far lessen the interchange of carbonic acid gas for oxygen in the lungs, as to keep the carbonaceous elements of the blood constantly in excess, and the play of vital affinity in the tissues, slightly retarded. A slow but certain accumulation of fatty tissue will be the result, with a corresponding impairment of the nervous and muscular vigor. Eventually, however, the tendency to fatty deposits will be manifested in the liver, kidneys, muscular structure of the heart, and even the coats of the blood-vessels, especially of the brain; and life will generally be terminated between the ages of forty-five and sixty years, by apoplexy, cardiac failure, or dropsy, induced by hepatic or renal disease.

This hasty review of the well-known pathological effects of alcoholic drinks, shows results strictly in accordance with the deductions from experiments and the observed facts of every day life.

Hence we are compelled to designate them as anæsthetic and sedative; anæsthetic to the nervous system, and sedative to the properties of the tissues.

As such they are capable of being used to fill a limited number of indications in the treatment of disease. And yet, there are other well-known agents in the materia medica that will meet the same indications equally well, or even better. So true do I deem this assertion, that for twenty years, I have not prescribed for internal use the amount of one pint of alcoholic drinks annually, including both hospital and private practice.

(*To be continued.*)

